# Supplementary material for: Randomized trial to compare acceptability of magnesium sulphate administration for preeclampsia and eclampsia: Springfusor pump versus standard of care
Source: PLoS One. 2024 Jun 12;19(6):e0286361. doi: 10.1371/journal.pone.0286361 (PMC11168672; doi:10.1371/journal.pone.0286361)
Supplement: S2 File — (DOCX) [file pone.0286361.s002.docx]

**Randomized trial to compare Magnesium Sulphate administration for preeclampsia and eclampsia: Springfusor versus standard approach**

Principal Investigator:

**Dr. Sam Ononge**

MBChB, MMed (Obstetrics & Gynaecology), PhD

Senior Lecturer Department of Obstetrics & Gynaecology

Co-Investigators:

**Dr Josaphat Byamugisha Kayogoza**

MBChB, MMed (Obstetrics & Gynaecology), PhD

Associate Professor Department of Obstetrics & Gynaecology

**Dr. Annettee Nakimuli**

MBChB, MMed (Obstetrics & Gynaecology), PhD

Associate Professor Department of Obstetrics & Gynaecology

**Dr. Hillary Bracken, MA, MHS, PhD**

Senior Director

**Dr. Thomas Easterling, MD**

Department of Obstetrics and Gynecology

Table of Contents

[Abstract 5](#_Toc497206426)

[Introduction 6](#_Toc497206427)

[Literature review 7](#_Toc497206428)

[Diagnosis of preeclampsia 7](#_Toc497206429)

[Management of preeclampsia 8](#_Toc497206430)

[Administration of magnesium sulphate 9](#_Toc497206431)

[Springfusor and flow control tube 10](#_Toc497206432)

[Statement of the problem 12](#_Toc497206433)

[Justification 13](#_Toc497206434)

[Research questions 14](#_Toc497206435)

[Methods 15](#_Toc497206436)

[Setting: 15](#_Toc497206437)

[Trial design: 16](#_Toc497206438)

[Study participants: 16](#_Toc497206439)

[Intervention 16](#_Toc497206440)

[Sample size 17](#_Toc497206441)

[Randomisation 17](#_Toc497206442)

[Trial flow chart 19](#_Toc497206443)

[Study procedure 19](#_Toc497206444)

[Adverse Events Reporting 20](#_Toc497206445)

[Outcome Measures 22](#_Toc497206446)

[Data management 24](#_Toc497206447)

[Safety and monitoring 26](#_Toc497206448)

[Ethical consideration 28](#_Toc497206449)

[**Informed Consent** 29](#_Toc497206450)

[Participant confidentiality 29](#_Toc497206451)

[Benefits to participants 29](#_Toc497206452)

[References 31](#_Toc497206453)

Abbreviations

BP Blood pressure

DSMB Data safety and monitoring board

gm grams

FCT Flow control tube

HELLP Hemolysis elevated liver enzymes Low platelet

IM Intramuscular

IV Intravenous

Ml millilitres

MFM Maternal foetal medicine

MgSO4 Magnesium sulphate

PE/E Preeclampsia/Eclampsia

SAE Severe adverse events

WHO World health organisation

Operational definition

1. Acceptability of the Springfusor: assessed using a likert scale ranging from 1 very acceptable, 2 acceptable, 3 neutral 4 unacceptable and 5 very unacceptable. Participants who scored 2 or less on the Likert scale are categorized as having considered springfusor acceptable. Participants who score 3 or more are considered to have found the Springfusor device unacceptable.
2. Safety of Springfusor: During the administration of magnesium sulphate, safety of use of Springfusor will be assessed by evaluating the possible occurrence of magnesium sulphate toxicity. This will be assessed by occurrence of any of the following; respiratory depression<16/min, respiratory arrest, depressed patellar reflex, liver failure, renal failure, cardiac arrest, and need for calcium gluconate.
3. Springfusor pump: Is a device powered by the potential energy stored within a spring that provides a constant force to the barrel of the loaded syringe.

# Abstract

**Introduction:** Magnesium Sulphate is the drug of choice for prevention and treatment of seizures in preeclampsia and eclampsia. It is administered parenterally by intravenous (IV) and or intramuscular (IM) routes. The IM regimen used in low resource settings, requires repeated painful injections which may be a barrier to optimal utilization whereby, there is frequent omission of some doses or increased interval between maintenance doses and low patient acceptability of magnesium. In addition, the IM regimen may potentially lead to abscess formation. There is a need to test an alternative way of providing Magnesium Sulphate that avoids the adverse events of IM regimens.

**Objective:** To assess the acceptability and safety of Springfusor device in the administration of magnesium sulphate in preeclampsia and eclampsia.

**Methods:** The study will be open label clinical randomized trail conducted at Mulago national referral and teaching hospital, where, 482 women diagnosed with preeclampsia and eclampsia will be randomized in blocks to either Springfusor device or standard of care for the administration of magnesium sulphate. Women in the Springfusor group will have their loading dose (4 gm of 50% Magnesium sulphate in 10 ml syringe administered over 20 minutes) and maintenance therapy (4 gm of 50% Magnesium sulphate in 10 ml syringe administered over 4 hours. The 4 gm maintenance dose will be repeated every 4 hours for 24 hours) of Magnesium sulphate through an IV infusion administered using a Springfusor pump. The control group will have Magnesium sulphate administered according to the Pritchard regimen (standard hospital practice). The Pritchard regimen involves administration of loading dose of 4 gm of 20% Magnesium sulphate IV over 15-20 minutes, immediately followed by 10 gm of 50% Magnesium sulphate IM (5gm on each buttock). The maintenance dose of 5 gm of 50% Magnesium sulphate IM every 4 hourly in alternate buttocks continued for 24 hours.

The primary outcome is the acceptability of administration of Magnesium sulphate using Springfusor assessed using a Likert scale. The other outcomes are discontinuation and complications in the two arms. Analysis will be intention to treat.

# Introduction

Preeclampsia is a disorder that presents with a raised blood pressure and proteinuria in pregnancy through to postnatal period [[1](#_ENREF_1)]. Globally, preeclampsia complicates approximately 2-8% of the pregnancies [[2](#_ENREF_2)] The presence of convulsions with preeclampsia indicates eclampsia, however, occasionally seizures may occur in the absence of hypertension and/or proteinuria . Preeclampsia and eclampsia (PE/E) are life-threatening for both the mother and the fetus. Preeclampsia and eclampsia are among the leading causes of maternal deaths and disability worldwide, more especially in the low resource setting [[2](#_ENREF_2)]. The World Health Organization (WHO) estimates that, 16% of the maternal deaths in low resource settings are due to PE/E [[3](#_ENREF_3)]. According to 2016 annual report of Mulago National teaching hospital, PE/E ranked second to hemorrhage as a specific, direct cause of maternal deaths, accounting for 21% of the maternal deaths (.

Magnesium Sulphate is the drug of choice for prevention and treatment of Eclampsia [[3](#_ENREF_3)]. It is administered parenterally by intravenous (IV) and or intramuscular (IM) routes. The IV regimen achieves more stable serum levels of Magnesium Sulphate but requires the use of electronic infusion pump for safe delivery. In low resource settings, electric infusion pumps are not available or feasible. The IM regimen used in low resource settings, requires repeated painful injections which may be a barrier to optimal utilization whereby, there is frequent omission of some doses or increased interval between maintenance doses and low acceptability of magnesium sulphate. In addition, the IM regimen may potentially lead to abscess formation. There is a need to develop an alternative way of providing Magnesium Sulphate that avoids the adverse events of IM regimens that can be used in low resource settings.

# Literature review

Preeclampsia is one of the hypertensive disorders that complicates approximately 2-8% of the pregnancies [[2](#_ENREF_2)]. It is one of the leading causes of maternal and neonatal morbidity and mortality worldwide [[4](#_ENREF_4)]. Preeclampsia may affect the liver, the kidney, blood coagulation and the brain. When there is delay in control of hypertension and or termination of pregnancy, preeclampsia may progress to placental insufficiency and maternal organ dysfunction.

The risk to the fetus is due to reduced or inadequate blood supply because of the damaged placenta, where it may lead to intrauterine growth restriction and fetal demise. In addition, management of preeclampsia may require women to have an early delivery (to protect the mother or fetus from injury), which leads to premature births. Severe preeclampsia may lead to seizures (eclampsia) [[5](#_ENREF_5)]. However, women can develop eclampsia without severe disease.

## Diagnosis of preeclampsia

Preeclampsia is a multisystem disease characterized by hypertension accompanied by proteinuria. The new onset of hypertension after 20 weeks of pregnancy (a systolic BP of >140 mmHg or a diastolic blood pressure >90 mmHg), with the new occurrence of proteinuria (>0.3g/24 hours) is the criteria for diagnosis of preeclampsia [[1](#_ENREF_1)]. Preeclampsia is usually classified as non-severe or severe [[3](#_ENREF_3)]. Progression to severe can be fast, unanticipated and fulminant. Preeclampsia is not considered severe until systolic or diastolic blood pressure exceed 160 mmHg or 110 mmHg respectively [[6](#_ENREF_6)]. The American College of Obstetricians and Gynaecologists recommend that the diagnosis of hypertension requires at least two measurements of blood pressure taken 4 hours apart, however when the blood pressures are severe, the diagnosis can be confirmed within minutes to enable timely antihypertensive therapy [[1](#_ENREF_1)]. It is also recommended that the diagnosis of proteinuria is when a 24 hour excretion equals or exceeds 300 mg/l. However, when a quick decision is require or the facility is not able to perform 24 hour protein estimation, a urine dipstick reading of 1+ is suggestive of proteinuria [[1](#_ENREF_1)].

Women with mild preeclampsia usually are asymptomatic. However those with severe preeclampsia may have headache, blurring of vision and abdominal pain (epigastric). Maternal organ dysfunction associated with severe preeclampsia may present with HELLP syndrome (hemolysis, elevated liver enzymes and low platelets), eclampsia, stroke and disseminated intravascular coagulation [[2](#_ENREF_2), [3](#_ENREF_3)]. Leeman and colleagues have indicated that the presence of the following are features of severe preeclampsia; elevated creatinine level (>1.1mg/dl), hepatic dysfunction (transaminase levels > 2 times upper limit of normal or right upper quadrant or epigastric pain, new onset of headache or visual disturbance, pulmonary oedema and or platelet count < 100x10^9^ per l [[6](#_ENREF_6)]

## Management of preeclampsia

Prevention of preeclampsia remains contentious and is an area of active research, particularly the use of anti-inflammatory and micronutrients in pregnancy. The anti-platelet agents like aspirin have be shown to reduce the risk of preeclampsia by 17% [[7](#_ENREF_7)]. On the other hand, calcium supplementation during pregnancy is shown to halve the risk of preeclampsia, but only in women with low dietary intake of calcium [[8](#_ENREF_8)].

The definitive treatment of preeclampsia is the delivery of the fetus and placenta. However, before and after delivery of the baby and placenta, the goal of management is to control the blood pressure to normal range and minimize the development of complications like eclampsia. The choice of anti-hypertensive to use for control of raised blood pressure is guided by the respective national guidelines and there are variety of antihypertensive drugs which are safe in pregnancy. For prevention and control of eclampsia, magnesium sulphate is the drug of choice[[3](#_ENREF_3)]. It has be shown to reduce the risk of eclampsia by over 50% [[9-11](#_ENREF_9)].

## Administration of magnesium sulphate

In the management of preeclampsia and eclampsia, magnesium sulphate therapy could be given by continuous IV infusion or by administering an IV bolus and IM doses for the loading dose followed by IM injections every 4 hours. The IV regimen was described by Zuspan, whereby a loading dose of 4 gm of Magnesium sulphate IV over 10-15 minutes is followed by an infusion of 1 gm/hour continued for 24 hours after the last seizure [[12](#_ENREF_12)]. In most health facilities especially in low resource settings, the rate of infusion is controlled manually. However, in high resource settings, the IV infusion is administered by a variety of programmable electronic pumps. These electronic pumps are expensive and require electricity or battery to run it, making it less appropriate in low resource settings. In low resource settings due to unavailability of the electronic pumps, the alternative regimen described by Pritchard is used [[13](#_ENREF_13)]. The Pritchard regimen involves administration of loading dose of 4 gm of 20% magnesium sulphate IV over 10-15 minutes, immediately followed by 10 gm of 50% magnesium sulphate IM (5gm on each buttock). The maintenance dose of 5 gm of 50% magnesium sulphate IM every 4 hourly in alternate buttocks continued for 24 hours after the last convulsion or delivery of the fetus, whichever is later. The IM injections are associated with pain, hot flushes, somnolence and abscess formation at the site of injection. A number of guidelines have recommended the addition of 1 ml of lignocaine to the IM solution to alleviate the pain. Other adverse events associated with magnesium sulphate are reduced patellar reflex, respiratory depression and oliguria. The concerns about the toxicity of magnesium sulphate has been reported to result in reluctance of some of the health professionals to administer it without the availability of antidote (Calcium gluconate). However a review by Smith et al point out that the incidence of severe side effects is very low[[14](#_ENREF_14)].

## Springfusor and flow control tube

The Springfusor and Flow Control Tube (FCT) are developed by Go Medical Industries Pty Ltd based in Australia [[15](#_ENREF_15)]. The Springfusor pump and FCT is intended to provide constant IV or subcutaneous (SC) infusions in either 10ml, 30ml or 50ml configuration at a variety of preset flow rates.


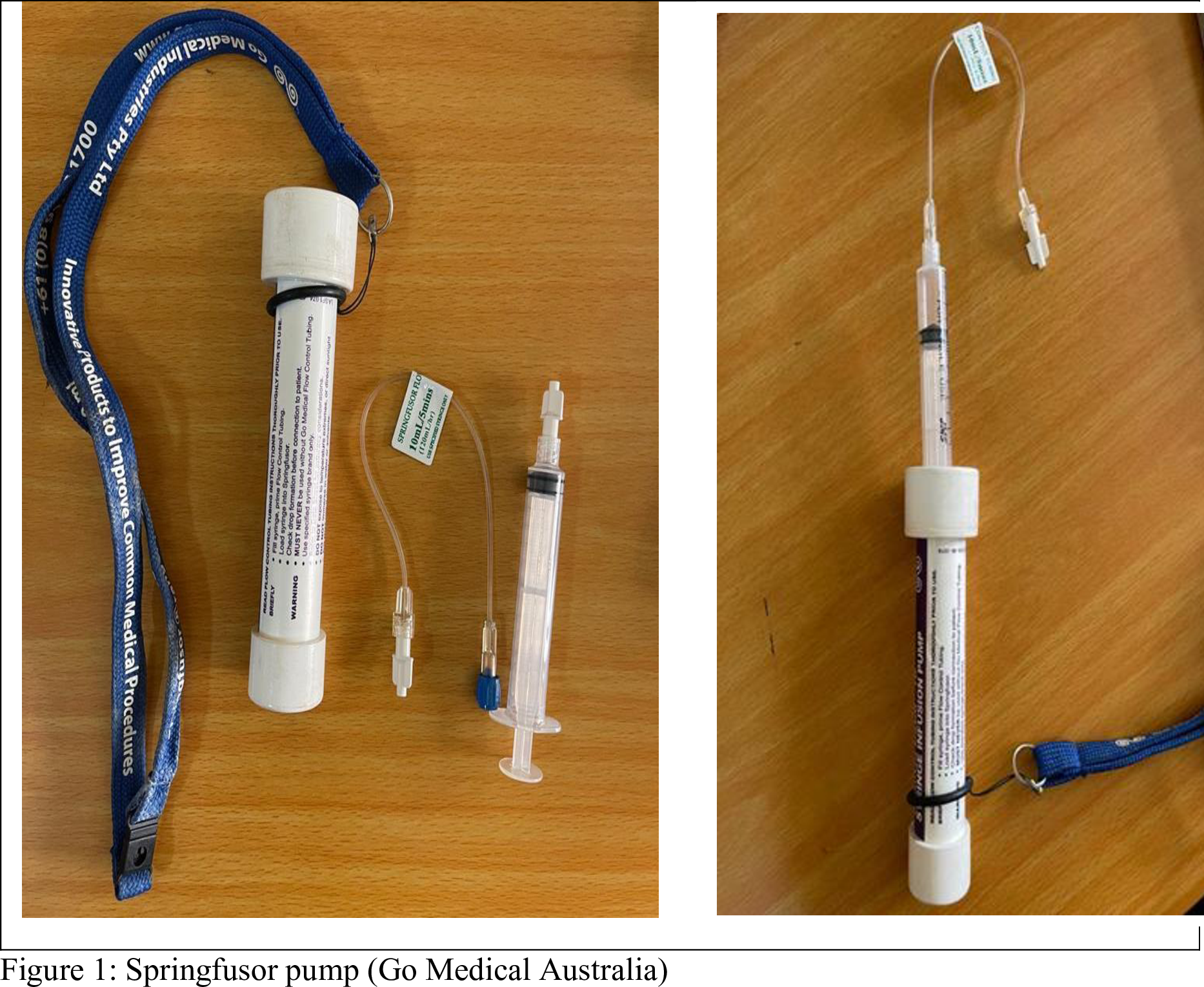
The Springfusor (Fig. 1) is powered by the potential energy stored within a spring at the heart of the device. The spring is compressed by the action of loading the Springfusor with the compatible syringe and FCT. The spring provides a constant force to the barrel of the loaded syringe. The flow rate is controlled by the FCT which offers consistent resistance to produce a steady flow. The FCT is a fine bore tube designed to provide a metered constant flow for IV infusion. FCTs has easy fitting to the patients cannula. The FCT exist in a variety of flow rates which enables the user to attain the desired output for exact IV delivery needs. While the Springfusor can be reused indefinitely on different patients, the FCT must be replaced after each use.

The Springfusor syringe infusion pump is a low cost, non-sterile, reusable pump that requires no external power source. It is simple to use and setup, and requires only minimal training to load and operate. It is lightweight, portable and therefore does not limit the mobility of the patient.

The Springfusor has been used to administer magnesium sulphate in the treatment of severe preeclampsia in India [[16](#_ENREF_16)]. In this trial, Mundle and colleagues compared the manually administered IV loading dose followed by maintenance therapy given by IM route of administration via a syringe, to a loading dose and maintenance therapy given through IV infusion administered by a Springfusor device. Though there were no difference in maternal and neonatal morbidity, the Springfusor had few side effects. Earlier in 1994, Freebairn et al in Australia compared the Spingfusor infusion device to intermittent bolus on administration of muscle relaxant. They were able to show that Springfusor provided a more constant level of paralysis compared to intermittent bolus administration [[17](#_ENREF_17)].

# Statement of the problem

Different anticonvulsants have been used for the prevention and treatment of seizures in preeclampsia and eclampsia, and they include; magnesium sulfate, phenytoin, diazepam, and “lytic cocktail” (usually chlorpromazine, promethazine, and pethidine). World health organization recommends magnesium sulphate as the anticonvulsive drug of choice for the treatment of severe preeclampsia and eclampsia [[3](#_ENREF_3)]. Magnesium sulphate is administered parenterally by intravenous (IV) and or intramuscular (IM) routes. The intravenous therapy is commonly administered following the Zuspan regimen that requires an initial loading dose of 4 gm of magnesium sulphate over 15-20 minutes, followed by 1-2 gm hourly maintenance dose continuing for 24 hours after the loading dose or the last eclamptic fit [[12](#_ENREF_12)]. This therapy is best delivered by infusion pumps. In most of the low resource settings magnesium sulphate administration follows the Pritchard regimen. The regimen is particularly complex and requires both the IV and IM administration. The loading dose of 4 gm is often delivered using an IV-push in which clinicians slowly inject magnesium sulphate with a syringe over 15-20 minutes. This is immediately followed by IM injection of 10 gm of magnesium sulphate into the gluteal muscles (5 gm on each buttock). The maintenance dose of 5 gm IM injection every 4 hourly for 24 hours. The Pritchard regimen requires different dilutions for IV and IM doses, and different doses for IV, IM, loading, and maintenance doses. This regimen requires a 20% dilution of magnesium sulphate for the IV loading dose, which necessitates the health providers to calculate the quantity of sterile water to add to the magnesium sulphate solution. In most settings, health providers do not encounter eclampsia very often; and when they do, trying to remember the complex regimen is challenging. In addition the Pritchard regimen requires administration of large, painful intramuscular (IM) injections (11 mL) every 4 hours for maintenance doses. The repeated IM injections can lead to risk for and development of abscess. Because of pain associated with the injection, some providers do not administer maintenance therapy and patients too, may discontinue the maintenance dose for the same reason. In addition the slow IV-push loading dose leads to inconsistent flow rates and is time-consuming, in setting with staff shortages. Furthermore, if magnesium toxicity occurs with IM regimen, the IM dose may not be reversed. The fear that toxicity may occur might lead providers to undertreat with magnesium sulphate. In the circumstances that low resources settings cannot afford electronic infusion pumps, there is a need to test alternative devices that can effectively deliver magnesium sulphate and safely, at cheaper cost, and acceptable to the patient and health provider. The Springfusor pump designed by Go Medical Industries is a promising alternative to Pritchard regimen and is designed to simplify continuous IV infusions. The Springfusor pump does not require electricity and its reusable. The aim of this study is to assess the acceptability and safety of Springfusor pump in delivery of magnesium sulphate for the treatment of preeclampsia and eclampsia.

## Justification

Preeclampsia and eclampsia (PE/E) are life-threatening for both the mother and the fetus. Ministry of Health guidelines recommends magnesium sulphate using Pritchard regimen for the prevention and control of seizures. Given the unavailability of electronic infusion pumps in low resource settings, and with the limitations of Pritchard regimen Springfusor pump is hoped to be an alternative infusion pump. It is cheaper than electronic infusion pumps, reusable and does not restrict the patient’s mobility. It can used in setting without electricity. In addition this pump could reduce the side effects associated with IM injections eg, pain, hot flushes and abscess formation. It is hoped that the Springfusor may improve the acceptability and continuation rate of magnesium sulphate dosing. The findings from this study will help determine if a Springfusor device can facilitate ease and safety of MgSO4 administration. If the Springfusor device is advantageous, then the results from this study can be used to argue for scale-up of this product, to ultimately expand access to prevention and control of seizures in women with preeclampsia. The results from this project will help the Ministry of Health to improve the care of women who experience high blood pressure during pregnancy. We hope that the Springfusor could be of benefit to women with high blood pressure who are to receive magnesium sulphate in that you have less side effects

## Research questions

1. Acceptability:
   1. What is the overall acceptability of Springfusor use among women with preeclampsia and eclampsia?
   2. What is the acceptability of the springfusor use for magnesium sulphate administration among health providers
   3. What is the level of pain associated with using Springfusor compared to the standard of care in administration of magnesium sulphate?
2. Effectiveness of Springfusor:
   1. What is the discontinuation rate of magnesium sulphate administration using a Springfusor pump compared to standard of care?
3. Safety
   1. What is rate of complications in the two arms?

Hypothesis

Use of Springfusor pump in the administration of magnesium sulphate in women admitted with preeclampsia and eclampsia is more acceptable than the hospital standard practice

Objectives

Primary objective

1. To compare the acceptability of the Springfusor pump to the standard of care in the administration of magnesium sulphate.

Secondary objective

1. To compare the proportion of complications between the Springfusor pump and the standard of care groups
2. To determine level of pain associated with using Springfusor compared to the standard of care?
3. To determine the discontinuation rate of administration of magnesium sulphate among women admitted with preeclampsia and eclampsia
4. To assess the reliability of Springfusor pump in the delivery of magnesium sulphate when used repeatedly
5. To assess the acceptability of the Springfusor pump for the administration of magnesium sulphate among the health providers.

# Methods

### Setting:

The study will be conducted at Mulago Hospital, a national referral and teaching hospital. The department of Obstetrics and Gynaecology is currently housed at Kawempe Hospital. The Maternal Fetal Unit (MFM) of the department admits approximately 2100 pregnant women per month. Among the MFM admissions, 7% of them are preeclamptic/eclamptic women. The women with preeclampsia and eclampsia are managed in two wards by carders of health workers ranging from senior consultants to intern doctors. The midwives are the ones who administer the medication.

### Trial design:

Randomized Open label Clinical Trial

### Study participants:

Inclusion criteria:

1. The study will include pregnant women 20+ weeks of gestation or delivered within 24 hours,
2. Presenting with preeclampsia and eclampsia i.e. have a raised blood pressure (systolic of >140 mmHg and diastolic > 90mmHg), proteinuria >1+.
3. Present within the study period
4. Consent to participate in the study.

Exclusion criteria:

1. Pregnant women or delivered within 24 hours who admitted with had received magnesium sulphate 24 hours prior to admission,
2. Has known allergy to magnesium sulphate and
3. Has elevated serum creatinine (>1.2 mg/dl). However the participants may be enrolled prior to the knowledge of serum creatinine, but withdrawn if the level is >1.2 mg/dl.

### Intervention

Women in the intervention arm (Springfusor group) will have their loading and maintenance therapy of magnesium sulphate using IV infusion administered using a Springfusor infusion pump. The loading dose will be 4 gm of 50% magnesium sulphate in 10 ml syringe administered over 20 mins. The maintenance dose of 4 gm of 50% magnesium sulphate in 10 ml will administered over 4 hours. The 4 gm dose will be repeated every 4 hours for 24 hours

Women in the control arm (Standard of care group) will have magnesium sulphate administered according to the standard hospital practice. The loading dose of is administered using an IV-push in which health worker manually and slowly inject 4 gm of 20% Magnesium sulphate in 20 ml syringe over 15-20 minutes. This is immediately followed by IM injection of 10 gm of 50% magnesium sulphate and 1 ml of lignocaine into the gluteal muscles (5 gm on each buttocks). The maintenance dose of 5 gm IM injection every 4 hourly for 24 hours. Over the 24 hour period the intravenous group (intervention) would have got 28 gm of magnesium sulphate while the intramuscular group (control) would have received 39 gms. The higher total dose in the control group is assuming a reduce bioavailability of intramuscular drug.

### Sample size

In the Mundle et al trial[[16](#_ENREF_16)], the Pritchard regimen (IM maintenance) the acceptability of magnesium sulphate was 31%. We are assuming that the women in Springfusor are likely to find magnesium sulphate administration more acceptable by 50%. With power of 90 and alpha of 0.05, we estimate that we need 219 women with preeclampsia in each arm. With approximately 10% non-response, we need 241 women in each arm.

### Randomisation

A biostatistician at the Clinical Epidemiology Unit of Makerere University College of Health Sciences and not involved in the clinical trial will develop allocation sequence using an online computer random number generator in different block sizes. Women with preeclampsia and eclampsia will be randomized to have their magnesium sulphate administered with Springfusor pump or the hospital standard of care. The study participants will be randomized only after they have qualified for the study and signed informed consent. Serially numbered sealed opaque envelopes (concealed allocation) containing the randomization group will be provided at site in a secure place within the ward. Trained research staff will open the envelope to determine the group assignment after the participant is enrolled, completed all the baseline assessment and it is time to allocate the intervention

Blinding: Because of the nature of the study, it is difficult to blind the study, the implementation of the allocation and measurement of the outcome.

### Trial flow chart

| 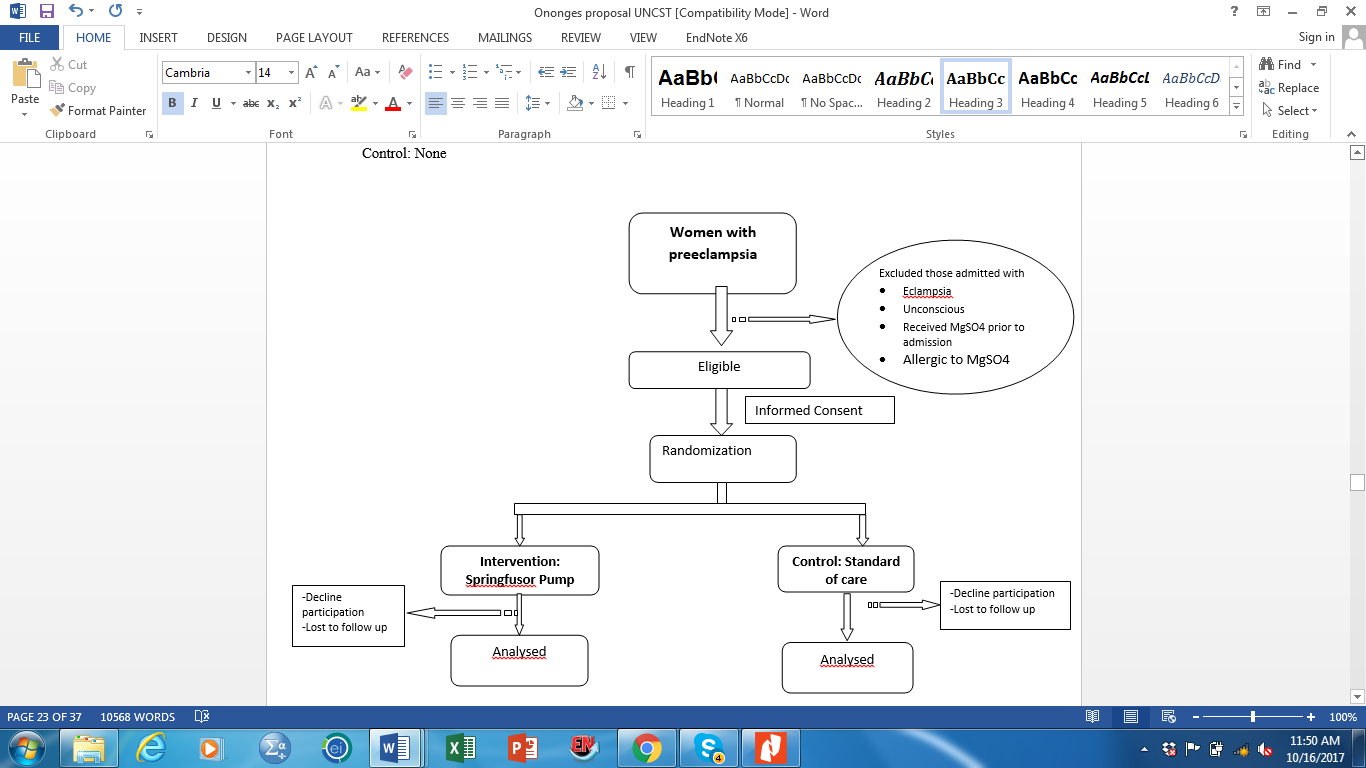  **Elevated creatinine level (>1.2 mg/dl)**  **eclampsia** |
| --- |

### Study procedure

Women with preeclampsia and eclampsia admitted at Mulago hospital maternal fetal unit will be screened and enrolled into the study if they meet the inclusion criteria. Both arms will receive the standard care as is currently given to women with preeclampsia in the health facilities; which includes management of high blood pressure, laboratory investigations (urine analysis, RFTs & LFTs) and delivery as planned by the attending physicians. Upon enrolment, study participants will receive the magnesium sulphate as per the randomization and any medication prescribed by the attending physician and received by the woman will be recorded in the CRF. The time it takes to administer the loading dose will be measured. Similarly the time it takes to administer the maintenance dose using the Springfusor will be measured using stop clock from the loading of the magnesium sulphate into device to completion of the medicine in the syringe.

The study participant’s respiratory rate will be monitored by the study midwives every 5 minutes during the loading dose (30 minutes). During the 24 hours maintenance dose, the participant’s respiratory rate, urine output and tendon reflexes will be monitored hourly. In addition, at four hourly interval, other side effects of magnesium sulphate like nausea, vomiting, flushing of the skin, muscle weakness, confusion and drowsiness will be noted. A report of pain and sign of inflammation will also be noted. The study participants will be followed till discharge and the women’s outcomes of the pregnancy will be reported. All women enrolled in the study will have an interview performed before discharge from the hospital. Study staff will administer a brief interview to capture information on the woman’s acceptability of magnesium sulphate using Likert scale, including the degree of pain experienced during administration of magnesium sulphate using a visual analogue scale. A standard operating procedure manual will be produced and provided to the study staff to enable adherence to the protocol.

### Adverse Events Reporting

**Definitions of an adverse event**

An adverse event (AE) is defined as "any untoward medical occurrence in a participant administered a pharmaceutical product that does not necessarily have a causal relationship with this treatment" Examples of adverse events include but are not limited to:

1. Worsening of conditions present after the intake of medicine (magnesium sulphate)
2. Events related or possibly related to concomitant medications
3. Hypersensitivity to magnesium sulphate
4. Signs and symptoms resulting from drug overdose
5. Any other significant complication

A serious adverse event (SAE) is defined as an experience that results in any of the following outcomes:

1. Death during the period of study follow-up
2. Life-threatening experience (one that puts a participant at immediate risk of death at the time of the event)
3. Prolonged hospitalization during the period of study follow-up
4. Persistent or significant disability or incapacity
5. Specific medical or surgical intervention to prevent one of the other serious outcomes listed in the definition.

**Reporting of serious adverse events**

For each serious adverse event identified and graded as moderate, severe or life threatening, a serious adverse event report form will be completed (SOMREC form).

The adverse events will be documented and submitted to the IRB with the annual report while the serious adverse events will be reported in 24 hrs and then full report within 5 working days. Guidelines for reporting of serious adverse events provided by the Makerere University School of Medicine Research and Ethics Committee, the Ugandan National Council for Science and Technology.

**Management of adverse events**

All adverse events regardless of the suspected causal relationship to the investigational product will reported to the PI. A physician attached to the study will treat the adverse event in consultation with the attending obstetrician on duty. Participant requiring more specialized care will be linked to care within or out of the hospital. The PI will assess the SAE causal relationship to the study device, follow up the participant until the event or sequelae has resolved or stabilized to a level acceptable to the IRB and data safety monitoring board.

#### Stopping rules

#### Stopping will be implemented if the use of the Springfusor for administration of magnesium sulphate becomes a national policy or if at the interim review, the acceptability of the Springfusor for the administration of magnesium sulphate is superior than the standard of care or, there significant complications in the Springfusor arm. However, during the accrual of the study, when the cases experiencing respiratory depression due to magnesium sulphate toxicity in the Springfusor arm exceeds 12.5%, an emergency DSMB meeting will be called to review if the study is to stop.

### Outcome Measures

Primary outcome:

- Acceptability of Springfusor for administration of magnesium sulphate will be assessed using a Likert scale ranging from one (very acceptable) to five (very unacceptable). The method of magnesium sulphate administration is regarded acceptable if its rated 1 and 2 on the likert scale. It is not acceptable if the ranking is three and above. Women who discontinue the method of administration by choice or due to side effects will be considered in group of unacceptable.

Secondary outcomes:

- *Rate of complications of preeclampsia and its management:* Proportion of preeclamptic women who developed magnesium sulphate toxicity, abnormal liver and renal function test as reported by the laboratory report, infection at the injection as reported by the clinician and maternal deaths
- Discontinuation: This will be assessed as study participants who do not completed doses of magnesium sulphate in 24 hour period of including the loading dose. Participants who discontinue the method of administration by choice, due to side effects or by health workers decision will be considered in group. This will be measured by the number of dose received for each arm. For the Pritchard arm (standard of care), the number of complete 6 doses equivalent to 29 gm (including loading dose) in 24 hours. In the Springfusor, the number of complete 6 doses equivalent to 24 gm (including loading dose) in 24 hours.
- *Level of pain:* The study participants will be asked to assess the severity of pain during the administration of magnesium sulphate using Visual analogue scale 1-7. The least (one) representing no pain and maximum (7) representing the worst pain imaginable.
- Safety of use of Springfusor will be assessed by evaluating the possible occurrence of magnesium sulphate toxicity. This will be assessed by occurrence of any of the following
  - respiratory depression<16/min,
  - respiratory arrest,
  - depressed patellar reflex,
  - liver failure, renal failure,
  - cardiac arrest,
  - a need for calcium gluconate.
- In addition, other side effects of magnesium sulphate like nausea, vomiting, flushing of the skin, muscle weakness, confusion and drowsiness will be noted.
- *Feasibility of Springfusor pump use: ⃰* The proportion of women who have a Springfusor used for administration of magnesium sulphate and experience some technical problem with its use, blockage of FCT, the number of attempts made prior to successful Springfusor placement, the number of failed Springfusor placement and the number of FCTs and spilled magnesium sulphate used will also be documented.
- The *reliability** of the Springfusor to administer magnesium sulphate in the prescribed time will assessed for the loading period and the maintenance period. The intra-correlation coefficient for the device will be calculated.

These outcomes will be compared between the women in the two arms. A *outcome will be reported only for women who will have magnesium sulphate administered using Springfusor

### Data management

**Data handling**

Case record forms will be provided for each participant. Participants will be identified by their study identification number on the case record form. Participant names will not be entered into the computerized database. All participant record forms will be kept in individual files in a secure filing cabinet. Only authorized study personnel will have access to these files. The case record forms will be cross checked by the PI and study coordinator. All corrections will be made on case record forms by striking through the incorrect entry with a single line and entering the correct information adjacent to it. The correction will be initialed and dated by the investigator.

Data will be transferred from the case record forms into a computerized database (Epi data version 3.1) by data entry personnel and will be doubly entered to verify accuracy of entry. For quality control, check programs will be written into the database to limit the entry of incorrect data and ensure entry of data into required fields.

**Data quality assurance**

A pilot study of 5 study participants on the use of Springfusor for the administration of magnesium sulphate will be done prior to implementation of the trial to get familiar with the device and test the study instruments. All members of the study team will be trained in the study protocol prior at the onset of the trial. The research assistants will complete case record forms. These forms will be reviewed by the study coordinator and PI for completeness and accuracy. Study group meetings will be conducted by the PI to assess progress of the study, address any difficulties, and provide performance feedback to the members of the study group. The study site will be open to review by the IRBs and or to inspection by appropriate regulatory authorities.

**Data analysis**

Data analysis will be performed by the PI. Descriptive statistics will be used to summarize baseline characteristics of study participants and assess if randomization was successful. The baseline characteristics will too give guidance as to which variables need to be adjusted for in the final analysis.

Acceptability and safety data will be evaluated using intention-to-treat (ITT) analysis. For comparing the maternal health outcome between intervention and control arms, the main analyses will look at the following variables;

1. Proportion of study participants who regarded magnesium sulphate administration using Springfusor or Pritchard route was acceptable. This will be compared using chi-square.
2. *Rate of complications of preeclampsia and its management:* Proportion of preeclamptic and eclamptic women who developed magnesium sulphate toxicity, abnormal liver and renal function test, infection at the injection and maternal deaths
3. The intensity of pain will be analysed using a ttest and presented as means and standard deviation
4. *Feasibility of Springfusor pump use:* The proportion of women who have a Springfusor used for the administration of magnesium sulphate and experience some technical problem with its use, blockage of FCT, the number of attempts made prior to successful Springfusor placement, the number of failed Springfusor placement and the number of FCTs used and spilled magnesium sulphate.
5. *The reliability of the springfusor:* The intra-class correlation coefficient (ICC) will be calculated. The closer the ICC to 1, the higher the reliability, ICC <0.4; the poor the reliability and ICC 0.4-0.75; the fair the reliability.

# Safety and monitoring

The following mechanisms will be employed to ensure the data quality and integrity of the study. An independent data and safety monitoring board (DSMB) will be established to review the data and make recommendations to investigators concerning study continuation and study modification. The main purpose of the DSMB is to ensure that the study participants are not exposed to undue risk. The DMSB will be called upon to review reported serious adverse events and provide advice on management of the study with relation to risk, and will help assure the continued scientific validity and merit of the study. An interim analysis will be conducted in the study when 2/3 of participants have been recruited. Based on the analysis and if deemed necessary, the DSMB will be asked to provide recommendations on study modification. All data analyzed by the DSMB will be reviewed in strict confidence.

The DSMB will consist of three professionals whose combined experience covers the fields of obstetrics and gynaecology, preeclampsia and eclampsia, biostatistics, and ethical conduct of human subject research. Qualifications for selection include: 1) Expertise in the fields of interest, 2) Experience in conduct of clinical trials and statistical knowledge, 3) independence from the direct management of the clinical trial, 4) no financial, scientific or other conflict of interest. Signed conflict of interest statements from each board member must be on the file at PI office prior to initiating DSMB activities.

Accountability log will be used to track the use of Springfusor devices and FCTs during the study. The product will be stored in accordance with the requirements of Springfusor. Any unused Springfusor and FCTs will be held at Mulago Hospital until final instructions are received from Go Medical Ltd.

**Conflict of interest**

The investigators declare no conflict of interest. The support for this study is provided by NURTURE program funded by NIH/Fogarty fellowship, which have not contributed to the design of the study and have relationship with the manufacturer of the Springfusor device. The Springfusor device will be provided by Go Medical industry Ltd, Australia at reduced rate and they too have not contributed to the design of the study. More information about Springfusor device can be viewed in the link: www.gomedical.com.au.

# Ethical consideration

This protocol and the informed consent documents, including any additional educational or recruitment material, will be reviewed and approved by Makerere University School of Medicine Research and Ethics Committee, Uganda National Council for Science and Technology before the trial begins. Any amendments or modifications to this material will also be reviewed and approved by the IRBs prior to implementation.

The process by which the information will be given and consent obtained will depend on the need for urgent clinical intervention and her physical, mental and emotional state. Also the availability and ability of a relative to make a decision on the woman’s behalf will have to be taken into consideration.

a) The woman is fully competent: the woman will be approached with the agreement of the primary health care worker at the time of diagnosis. An information sheet will be provided and written consent obtained.

b) The woman mental state is impaired (eg eclampsia) and relative is available: The relative will be provided with the information. Opportunity for questions should be given and written consent obtained. If the relative is unable to read or write, then the information sheet may be read to her/him and thumb print made on the consent. If the relative is not available, then doctor or midwife may be asked to consent as a professional representative. For women enrolled under such emergency consent procedure, the woman or her relative should be informed about the study as soon as she is stable and asked to consent for continuation of any trial procedure. This consenting process will occur once the woman is stable following treatment of the seizure and she is conscious, and she will afford the opportunity to ask any questions she has about her care. Women have the right to refuse or discontinue being in the study.

## **Informed Consent**

The study midwives will obtain an informed consent from all participants enrolled into this study or the available relative as indicated above. All information that is given to the participant will be in a language that is understandable to her. If the woman is willing to participate she will be asked to read and sign the informed consent form. If the woman is unable to read, study staff will read and explain the form to the women, and the consent will be indicated by a mark, such as the woman’s thumbprint.

A copy of the signed consent form will be given to each participant. The original signed consent form for each participant will be kept at Mulago Hospital and in the participant’s study files separate from the CRFs. Study staff will be trained in the informed consent process. Women who decline to participate in the study will receive standard of care available at the site.

## Participant confidentiality

All study staff will protect the confidentiality of participants’ informed consent documents with participants’ names will be stored separately from the study CRFs. No participant personal identifiers will appear on the CRFs or publication

## Benefits to participants

Participants assigned to the Springfusor device may experience a less painful at the site of injection. However, it is possible the standard approach is equal on these measures; in this case, no participants will benefit directly from this study.

The findings from this study will help determine if a Springfusor device can facilitate ease and safety of magnesium sulphate administration. If the Springfusor device is advantageous, then the results from this study can be used to argue for scale-up of this product, to ultimately expand access to prevention and control of seizures in women with preeclampsia.

Women who participate in this study will not have to pay for Springfusor or FCTs.

# References

1. *Report of the National High Blood Pressure Education Program Working Group on High Blood Pressure in Pregnancy.* Am J Obstet Gynecol, 2000. **183**(1): p. S1-S22.

2. Duley, L., *The global impact of pre-eclampsia and eclampsia.* Semin Perinatol, 2009. **33**(3): p. 130-7.

3. *WHO Recommendations for Prevention and Treatment of Pre-Eclampsia and Eclampsia*. 2011: Geneva.

4. Collaborators, G.B.D.M.M., *Global, regional, and national levels of maternal mortality, 1990-2015: a systematic analysis for the Global Burden of Disease Study 2015.* Lancet, 2016. **388**(10053): p. 1775-1812.

5. Salam, R.A., et al., *Diagnosis and management of preeclampsia in community settings in low and middle-income countries.* J Family Med Prim Care, 2015. **4**(4): p. 501-6.

6. Leeman, L., L.T. Dresang, and P. Fontaine, *Hypertensive Disorders of Pregnancy.* Am Fam Physician, 2016. **93**(2): p. 121-7.

7. Duley, L., et al., *Antiplatelet agents for preventing pre-eclampsia and its complications.* Cochrane Database Syst Rev, 2007(2): p. CD004659.

8. Hofmeyr, G.J., et al., *Calcium supplementation during pregnancy for preventing hypertensive disorders and related problems.* Cochrane Database Syst Rev, 2014(6): p. CD001059.

9. Duley, L., et al., *Magnesium sulphate and other anticonvulsants for women with pre-eclampsia.* Cochrane Database Syst Rev, 2010(11): p. CD000025.

10. *Which anticonvulsant for women with eclampsia? Evidence from the Collaborative Eclampsia Trial.* Lancet, 1995. **345**(8963): p. 1455-63.

11. Altman, D., et al., *Do women with pre-eclampsia, and their babies, benefit from magnesium sulphate? The Magpie Trial: a randomised placebo-controlled trial.* Lancet, 2002. **359**(9321): p. 1877-90.

12. Zuspan, F.P., *Problems encountered in the treatment of pregnancy-induced hypertension. A point of view.* Am J Obstet Gynecol, 1978. **131**(6): p. 591-7.

13. Pritchard, J.A., *The use of the magnesium ion in the management of eclamptogenic toxemias.* Surg Gynecol Obstet, 1955. **100**(2): p. 131-40.

14. Smith, J.M., et al., *An integrative review of the side effects related to the use of magnesium sulfate for pre-eclampsia and eclampsia management.* BMC Pregnancy Childbirth, 2013. **13**: p. 34.

15. *Go Medicals Industries Pty Ltd. Innovative Products to improve common medical procedures.* <http://www.gomedical.com.au/products/infusion/springfusor-fct> **Accessed September 25, 2017**.

16. Mundle, S., et al., *Treatment approaches for preeclampsia in low-resource settings: A randomized trial of the Springfusor pump for delivery of magnesium sulfate.* Pregnancy Hypertens, 2012. **2**(1): p. 32-8.

17. Freebairn, R., et al., *A double-blind comparison of vecuronium administered by the Springfusor infusion device to vecuronium by intermittent bolus injection in critically ill adult patients.* Anaesth Intensive Care, 1994. **22**(5): p. 580-5.
