# Supplementary material for: Randomized trial to compare acceptability of magnesium sulphate administration for preeclampsia and eclampsia: Springfusor pump versus standard of care
Source: PLoS One. 2024 Jun 12;19(6):e0286361. doi: 10.1371/journal.pone.0286361 (PMC11168672; doi:10.1371/journal.pone.0286361)
Supplement: Attachment — Submitted filename: Response to reviewers.docx [file pone.0286361.s004.docx]

Dear Reviewers,

Thank you once more for the contrastive comments and guidance. Below are Point-by-point responses to the concerns/queries raised.

| Query/Revision | Response in track changes |
| --- | --- |
| .1- Line 50, Delete this sentence, “baseline characteristics were similar in both arms”, this is an expected result in randomized trials that always needs to be checking, it is not specific of your study. | Thank you. Deleted (line 50) |
| 2.- Line 114, I think the authors misunderstood my previous comment regarding the objective. I was not asking about the definition of acceptability I am asking about the main aim. Do you want to estimate the percentage of women that consider Springfusor as acceptable? Or do you want to demonstrate the superiority of Springfusor, in terms of acceptability, compared to standard of care? In my opinion your objective is the later, therefore you should change it. | Thank you for the clarification. Edited as The objective of this study was to demonstrate that using Springfusor pump for intravenous delivery of magnesium sulphate in the treatment of preeclampsia and eclampsia is more safer and acceptable than the standard of care (lines 114-117) |
| 3.- Line 192, delete “to minimize the risk of type II error, you are fixing it at 10%. | Deleted (line 193) |
| 4.- Line 192, As I mentioned in point 2, sample size justification, objectives, statistical methodologies, and results must be in line. Reading this Section, it is clear that authors want to demonstrate the superiority of Springfusor compare to standard of care, but the name of the test used to do it, needs to be included, because the sample size depends on the approach. | The test is a difference in proportion and we have revised the statement to read “To demonstrate a 50% difference in the proportion of women who reported administration of mgso4 was acceptable between those who used springfusor and standard of care, we need to have a sample size of 241 in each arm.” (lines 195-199) |
| 5.-Line 212, change “The difference was considered statistically significant at p<0.05” by “p<0.05 was set to consider statistical significance” because it refers not only to the difference but also to any hypothesis. | Thank you. Changed to “The statistically significant was set at p<0.05.” (lines 216-217) |
| 6.- Line 236, Table 1, delete % from the first row. | Done |
| 7.- Line 236, Maybe “Particulars” is not the best denomination | Deleted |
| 8.- Line 240, Delete “p=003” | Done (line 244 |
| 9.- Line 248, change “4(CI: 4-5)” and “2(CI:2-2)” by 4(IQR: 4-5)” and “2(IQR:2-2)” | Done (lines 252-253) |
| 10.- Line 250, change “96% vs 61%” by “96.2% vs 61.4%” | Done (line 254) |
| 11.- Line 253, change “96.5” by “95.7” | Thank you. Done. (line 257) |
| 12.- Line 254, Table 3, delete 95% CI in the first row | Done |
| 13.- Line 254, Table 3, change CI for the median pain score by IQR and add CI in the rest of rows | Thank you Done |
| 14.- Line 254, Table 3, consider reviewing this sentence “would use it in future she got raised BP in next pregnancy” | Revised to read “Would consider using it again in future if she experienced a raised BP during her next pregnancy” |
| 15.- Line 255, add IQR=interquartile range | Thank you done (line 260) |
| 16.- Line 259, Table 4, As I said in my previous review, please use the same name in all tables when referring to the standard (or control) arm and intervention (Springfusor), besides sometime arms are used, in others they are denoted as groups… | Thank you . corrected |
| 17.-Line 259, Table 4, check this information, p-value=0.863 obtained from a Fisher test, this is not possible because this test cannot be used with more than two categories. | Thank you for that correction. Used Chi square |
| 18, Line 259, Table 4, check this information “Pain score …n(%)” if you are comparing percentages then Mann Whitney test is not appropriated | Thank you. Corrected |
| 19.- Line 260, Change Fishers by Fisher`s, (if you keep this test) | Changed |
|  |  |
|  |  |

Thanks
