# Supplementary material for: Randomized trial to compare acceptability of magnesium sulphate administration for preeclampsia and eclampsia: Springfusor pump versus standard of care
Source: PLoS One. 2024 Jun 12;19(6):e0286361. doi: 10.1371/journal.pone.0286361 (PMC11168672; doi:10.1371/journal.pone.0286361)
Supplement: Attachment — Submitted filename: Response to reviewers.docx [file pone.0286361.s005.docx]

Dear Reviewer,

I am so grateful for the guidance. Below are Point-by-point responses to the recommendations.

| Query/Revision | Response in track changes |
| --- | --- |
| 1. Line 197 : Please change this sentence: To demonstrate a 50% difference in the proportion of women who reported administration of mgso4 was acceptable between those who used Springfusor and standard of care, by To detect an absolute increase of 50 percent points in the proportion of women who reported administration of mgso4 was acceptable between those who used Springfusor and those who used standard of care, | Thank you  We have changed |
| 2. In Table 5 and Table 6 it is better not to include p-values | Deleted |
|  |  |

Thank you so much
